# Molecular and Phenotypic Characteristics of* Escherichia coli* Isolates from Farmed Minks in Zhucheng, China

**DOI:** 10.1155/2019/3917841

**Published:** 2019-06-27

**Authors:** Jianhua Qiu, Zhiyu Jiang, Zijing Ju, Xiaonan Zhao, Jie Yang, Huijun Guo, Shuhong Sun

**Affiliations:** College of Animal Science and Technology, Shandong Provincial Key Laboratory of Animal Biotechnology and Disease Control and Prevention, Shandong Provincial Engineering Technology Research Center of Animal Disease Control and Prevention, Shandong Agricultural University, Daizong Street 61, Tai'an 271018, China

## Abstract

In this study, the prevalence, phenotypes, and clonal relationships of* Escherichia coli *(*E. coli*) strains isolated from minks were investigated. In July 2017, a total of 62 fresh faecal swab samples were randomly collected from one large-scale mink farm in Zhucheng, Shandong Province, China. In all the samples, 50* E. coli* strains were isolated and then assigned to serotyping, antimicrobial susceptibility test, detection of antimicrobial resistance genes and the Class 1 integrons, and multilocus sequence typing (MLST). Four pathogenic serotypes were identified among all the isolates, while the most common serotype was enterohemorrhagic* E. coli* O104:H4 (6.0 %). Antimicrobial sensitivity testing revealed that most isolates were susceptible to cefoxitin (96.0 %) and amikacin (82.0 %), while most isolates were resistant to ampicillin (92.0 %) and tetracycline (90.0 %). An analysis of the nucleotide sequences revealed that 7 isolates (14.0%) carried 4 types of Class 1 integron cassette, including* dfrA27+aadA2+qnrA *(57.1%),* dfrA17+aadA5* (14.3%),* dfrA12+aadA2* (14.3%), and* dfrA1+aadA1 *(14.3%). PCR screening showed that 14 antibiotic resistance genes were presented in 50 isolates, while the most prevalent resistance gene was* qnrS*, which was detected in 60.0 % of isolates, followed by* sul2* (40.0%) and* oqxA* (38.0%). MLST analysis showed that 32 sequence types (STs) were identified, while ST46 was the predominant genotype among all isolates. Clonal complex 3 (CC3) was dominant. Compared with 340 human* E. coli* STs reported in China, the ST10 clonal complex, known as the largest human clonal complex, was also found in the 50 mink* E. coli *isolates. Meanwhile, mink-derived strain ST206 formed a new clonal complex, CC206, which was different from human ST strains. Our results showed that farmed minks could be reservoirs of antimicrobial-resistant* E. coli* with Class 1 integron cassettes and resistance genes, which were likely to pose a threat to public health. Therefore, continuous inspections and monitoring of* E. coli* in minks are essential for detecting and controlling emerging* E. coli* with different serovars as well as antibiotic resistance.

## 1. Introduction

Since the advent of antibiotics, it had made great contributions to both human and animal health. Antibiotics that can kill or inhibit the growth of microorganisms have been used to treat a wide range of infectious diseases caused by bacteria, in livestock, poultry, wild, or fur animals (such as minks) and humans. Antibiotics play a very important role in dealing with pathogenic microorganism infections as well as in reducing morbidity and mortality [1, 2].

According to the differences in pathogenicity,* E. coli* can be classified into commensal* E. coli *and pathogenic* E. coli* [3]. Pathogenic* E. coli* may cause enteritis, urinary tract urethritis, and other diseases in warm-blooded animals [4, 5]. Some* E. coli* strains are potentially pathogenic, including enterotoxigenic* E. coli* (ETEC), enterohemorrhagic* E. coli* (EHEC), enteropathogenic* E. coli* (EPEC), enteroinvasive* E. coli* (EIEC), and enteroaggregative* E. coli* (EAEC), which may cause intestinal or urologic diseases [4, 5].

Antibiotics have long been considered as the first line of defense to prevent pathogenic* E. coli* infections. The treatment of pathogenic* E. coli* infections is becoming complicated because of the rapid emergence and dissemination of antibiotic-resistant strains, which may consequently result in an increasing number of clinical treatment failures in bacterial-mediated diseases and further threaten public health with the possibility of transmission to humans through aerosols, environmental contact or other methods [2, 6]. The level of antibiotic resistance among pathogenic and commensal* E. coli *has steadily increased and has become a global health concern [7, 8].

There are many resistance mechanisms underlying the emergence and prevalence of antimicrobial resistance. The acquisition of resistance genes through a mechanism involving mobile genetic elements, such as plasmids and transposons, is considered a major contributor to antimicrobial resistance [9, 10]. A previous study also demonstrated that* E. coli *can carry resistance plasmids and can easily acquire resistance transfer genes [9, 10]. The spread of antibiotic resistance among bacteria is mainly attributed to integrons. Integrons are DNA elements that are associated with the prevalence and horizontal transmission of antibiotic resistance [11–13]. Integrons can capture, express, and exchange gene cassettes and then convert them to functional genes [11, 14].

*E. coli* may cause severe morbidity and mortality in fur animals such as minks. In recent years, several types of antibiotics have been used in Shandong Province to treat bacterial diseases, resulting in repercussions for bacterial antibiotic resistance [15, 16]. To the best of our knowledge, in contrast to* E. coli* in humans, livestock, and poultry, there is still a lack of data about the antimicrobial resistance mechanisms of* E. coli* from minks in China. Therefore, in our study, 62 faecal samples were collected from mink farms in the Zhucheng area, and then* E. coli *strains were isolated for serotyping, antimicrobial susceptibility testing, detection of the Class 1 integron cassette, and resistance genes to provide information and guidance for the rational use of antibiotics in minks.

## 2. Materials and Methods

### 2.1. Sampling and Isolation

In July 2017, 62 faecal swabs were randomly collected from healthy minks in large-scale mink farms in the Zhucheng area in Shandong Province, China. Mink farms were chosen based on their scale with the requirement of the breeding stock being > 10000 heads. Ethical approval was not required for the study because the sampling process did not harm the minks. All the faecal samples were placed into an ice box and transferred to our laboratory within 6 h for further bacteriological analysis.

Isolation and identification of* E. coli* were performed according to the previously described method [17] with some slight modifications. Briefly, faecal swab samples were transferred to sterile culture tubes containing 4 mL of Luria-Bertani (LB) broth and mixed vigorously (220 r/min) at 37°C for 8 h. After enrichment, a loop of LB broth culture was streaked onto eosin-methylene blue medium (EMB) agar and incubated at 37°C for 24 h. Colonies showing a metallic sheen were considered presumptive* E. coli *isolates, and positive colonies were chosen for further biochemical identification using the API 20E system (Sysmex-bioMerieux, Tokyo, Japan).

### 2.2. Serotyping

To identify the* E. coli* serotype, all the positive strains were serotyped by* E. coli* diagnostic serum according to the manufacturer's instructions. The diagnostic serum kit consisted of EPEC diagnostic serum, EIEC diagnostic serum, ETEC diagnostic serum and EHEC (O104 and 0157) diagnostic serum, and* E. coli* H4 and H7 diagnostic serum (Tianrun Bio-Pharmaceutical, Ningbo, China). Briefly, the principle of the* E. coli* serotyping method was as follows: the diagnostic serum was directly mixed with a bacterial suspension on a slide, the specific agglutination reaction between antigens and the corresponding serum was observed, and then the serotype was determined.

### 2.3. Antimicrobial Susceptibility Testing

The Kirby-Bauer disk diffusion method, as described by the Clinical and Laboratory Standard Institute [18], was used in this study to test the susceptibility of* E. coli* to 14 commonly used antibiotics, including ampicillin (AMP, 10 *μ*g), amikacin (AMI, 30 *μ*g), ciprofloxacin (CIP, 5 *μ*g), nalidixic acid (NAL, 30 *μ*g), tobramycin (TOB, 10 *μ*g), ceftriaxone (CRO, 30 *μ*g), florfenicol (FFN, 30 *μ*g), tetracycline (TET, 30 *μ*g), gentamicin (GEN, 10 *μ*g), sulfamethoxazole (SXT, 25 *μ*g), chloramphenicol (CHL, 30 *μ*g), Augmentin (AUG, amoxicillin: clavulanate potassium = 20 *μ*g: 10 *μ*g), ceftazidime (CTA, 30 *μ*g), and cefoxitin (COX, 30 *μ*g).

*E. coli *(ATCC 25922) and Klebsiella pneumoniae (ATCC 700603) were used as the control strains.* E. coli* isolates that were resistant to more than three classes of antimicrobials were defined as multidrug resistance (MDR) isolates.

### 2.4. Statistical Analysis

We analysed the correlation between drug resistance phenotypes and drug resistance genes of 50 isolates. The statistical analyses were performed using SAS V8 software (SAS Inc., Raleigh, N Car, USA), employing the chi-square test. P values less than 0.05 were considered statistically significant.

### 2.5. Detection of the Class 1 Integron Cassette and Resistance Genes

*E. coli *isolates were characterized at the molecular level to detect their antimicrobial resistance mechanisms. Bacterial DNA was extracted using a TIANamp Bacterial DNA Kit (Tiangen, Beijing, China) according to the manufacturer's instructions. Gene cassette regions within the variable region of Class 1 integrons were detected via polymerase chain reaction (PCR). Primers were synthesized according to the references [19], and the sequences are shown in Table 1. The amplification consisted of an initial denaturation at 94°C for 5 min, 30 cycles of denaturation at 94°C for 60 s, annealing at 56°C for 55 s, and extension at 68°C for 6 min. A final extension for 10 min at 72°C was also applied. The PCR products were purified and then sequenced (Invitrogen, Beijing, China). The obtained DNA sequences were compared with those in GenBank using the Basic Local Alignment Search Tool (BLAST).

Twenty-four pairs of specific primers were designed according to known sequences and references in GenBank [20–28]. The primers used for PCR and the respective amplification lengths are shown in Table 1. The presence of antibiotic resistance genes in 50* E. coli *strains was analysed by PCR and sequencing.

### 2.6. Multilocus Sequence Typing (MLST)

MLST analysis was performed using seven pairs of primers described at http://bitbucket.org/enterobase/enterobase-web/wiki/ecoli%20MLST%20Legacy%20Info%20RST to detect seven housekeeping genes (adk, icd, mdh, gyrB, purA, recA, and fumC). PCR was performed, and the amplification products were sequenced by Shanghai Sangon Biotech Co., Ltd. DNA Star software and BLAST (Basic Local Alignment Search Tool) were used for sequence analysis and comparison. Subsequently, the comparison results were submitted to the Pasteur online database (http://enterobase.warwick.ac.uk/species/ecoli/allele_st_search), and the sequence type (ST) of each strain was determined.

Nucleotide sequences of* E. coli* O157:H7 str. Sakai (NCBI Reference Sequence: BA000007, ST11) [29] were obtained from the NCBI repository and included in subsequent analyses for comparison. To analyse the distribution of STs in free-range mink farms, a minimum spanning tree was generated using BioNumerics software, version 7.6 (Applied Maths, Kortrijk, Belgium).

In order to determine the clonal and epidemiological relationships as well as the formation of clonal complexes (CCs), genetic similarity diagram analysis was performed by the program eBURSTv3 [30] (http://eburst.mlst.net/). Genetic similarity diagrams can demonstrate the genetic relationship among bacteria through their respective ST analysis. Then, the bacteria can be grouped into single locus variants (SLVs), double locus variants (DLVs), and triple locus variants (TLVs) or be present in isolation (singletons). If the STs exist and there is an allele difference among the ST types, the diagram also allows verification of the grouping of STs, representing the CC [31].

In order to examine the relatedness between strains and STs at the sequence-level resolution, concatenated sequence data of each distinct representative ST were imported into the MEGA 6.0 software package [http://www.megasoftware.net/]. Through complete deletion of alignment gaps, a total of 3423 positions were used in each concatenated sequence as a data set for phylogeny calculations. An evolutionary phylogeny was constructed in MEGA 6 using the maximum composite likelihood (MCL) to estimate evolutionary distances, and the topology was validated by bootstrapping (1000 replicates) [32–34]. To establish evolutionary relevance,* E. coli* strain 11 (ST11) was used as the tree root. The optimum tree generated was condensed where the bootstrap support for the clustering of taxa was <50 % of the replicates [35].

In order to analyse the strain resistance phenotype and strain resistance gene relatedness in the phylogenetic tree, a phylogenetic tree was introduced into the EvolView software package [http://www.evolgenius.info/evolview/#login].

## 3. Results

### 3.1. Isolation and Serotyping of* E. coli*

In this study, a total of 50 nonduplicate* E. coli* isolates (50/62, 80.7 %) were recovered from mink faecal samples. The serological results showed that the most prevalent serovars were EHEC (3/50, 6.0 %) and ETEC (3/50, 6.0 %), including O104:H4 (3/50, 6.0 %), O20:K17 (2/50, 4.0 %) and O9:K9 (1/50, 2.0 %) (Table 2), while no EPEC was found among these* E. coli* isolates (0/50). EIEC accounted for 2.0 % (1/50), ETEC accounted for 6.0 % (3/50), and EHEC accounted for 6.0 % (3/50) of the isolates.

### 3.2. Antimicrobial Susceptibility Testing

**Figure 1:**
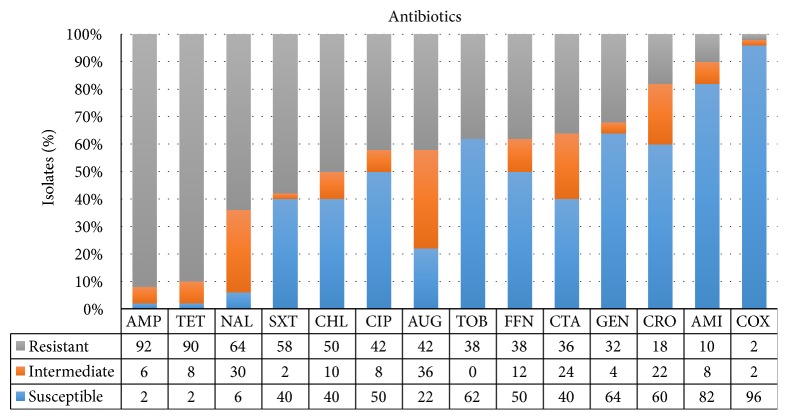
Antimicrobial resistance phenotypes of 50* E. coli* isolates.

The results of the antimicrobial susceptibility test for the 50* E. coli *isolates are shown in Figure 1. Most of the strains were susceptible to COX (48/50, 96.0 %) and AMI (41/50, 82.0 %), while most isolates were resistant to AMP (46/50, 92.0 %) and TET (45/50, 90.0 %). In addition, 43 isolates (43/50, 86.0 %) were MDR. The most prevalent resistance profile was AMP-SXT-TET (6.0 %) (Figure 4).

### 3.3. Characterization of Class 1 Integron Structure and Antimicrobial Resistance Genes

Among the 50* E. coli *isolates recovered from minks, 7 isolates (7/50, 14.0 %) carried Class 1 integrons cassettes, which contained four types of resistance gene cassette, including* dfrA27+ aadA2 + qnrA *(4/7, 57.1 %),* dfrA17 + aadA5 *(1/7, 14.3 %),* dfrA12 + aadA2 *(1/7, 14.3 %), and* dfrA1+ aadA1 *(1/7, 14.3 %) (Table 2). The isolates carrying the gene cassette were multidrug resistant, and the resistance genes qnrs and sul were present (Figure 4).

Fourteen resistance genes were detected in 50 isolates, and most isolates carried* qnrS* (30/50, 60.0 %), followed by* sul2* (20/50, 40.0 %) and* oqxA* (19/50, 38.0 %), while *bla*_SHV_, *bla*_CMY-2_,* aac(3)-I, aac(3)-III*,* Ant(2*′),* qnrA*,* qnrC*,* tetA*,* tetB*, and* stcM* genes were not found. Among the 50 isolates, two strains did not carry resistance genes, and one strain carried 9 resistance genes (Figure 4).

The statistical analysis showed that there was a positive correlation between the tobramycin/gentamicin resistance spectrum and the presence of the* aac(3)-II* gene (P<0.01). The resistance rate to tobramycin and gentamicin of strains carrying the* aac(3)-II* gene was 64.29 %, and for those without the* aac(3)-II *gene, the resistance rate to tobramycin and gentamicin was 25 % and 16.67 %, respectively.

### 3.4. MLST

Based on the MLST analysis results, 50* E. coli* strains were classified into 31 different sequence types (STs); ST46 (8/50, 16 %) was the most frequent ST, followed by ST398 (4/50, 8 %) and ST10 (3/50, 6 %). Each of the following STs accounted for 4 % (2/50): ST540, ST1434, ST710, ST226, ST206, and ST48. The other strains were individually classified into 22 different STs (Table 2).

**Figure 2:**
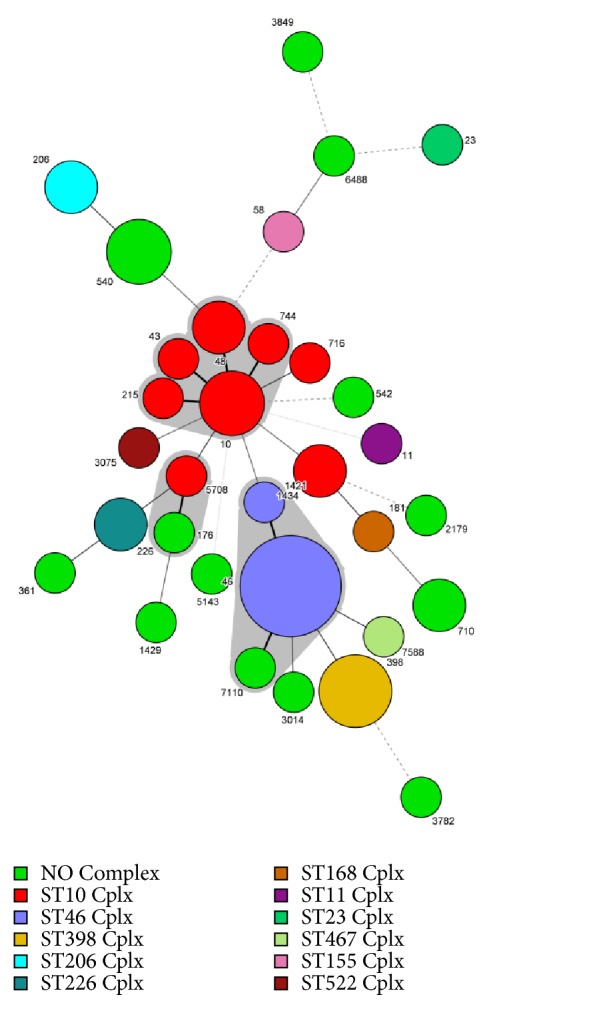
Minimum spanning tree analysis of* E. coli* isolated from free-range mink farms.* Note*. Each circle represents one ST, and the area of the circle corresponds to the number of isolates. The colour of the circle indicates the clonal complex to which the isolate belongs. The grey region indicates that strains of isolates belong to a clonal complex. ST11 is included as a reference.

Eighteen of the 31 ST genotypes originated from 10 clonal complexes, and the remaining 13 ST types did not have clonal complexes within the BURST algorithm. Ten different STs of the 50* E. coli* strains formed three clonal complexes, CC10, CC46, and CC176. Among the three clonal complexes, CC10 was the largest, containing eight isolates, consisting of ST10, ST43, ST48, ST215, and ST744 in ST10 Cplx. The second largest clonal complex was CC46, which contained ten isolates, consisting of ST46 and ST1421 in ST10 Cplx and ST7110 without a clonal complex. The minimum clonal complex was CC176, consisting of ST5708 in ST10 Cplx and ST176 without a clonal complex (Figure 2).

**Figure 3:**
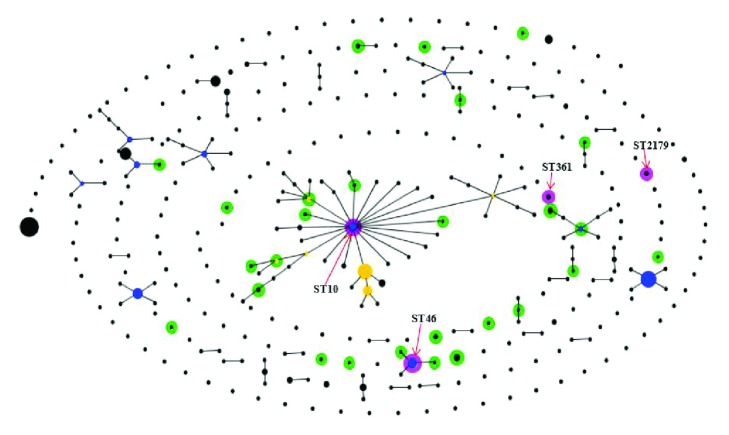
eBURST diagram generated by comparing the isolated strains dataset with the* E. coli* MLST database from human strains in China.* Note. *The* E. coli* MLST database from human strains in China was the reference dataset. STs in the profiles window are coloured differentially dependent on their membership of the two datasets. In pink, STs found in both datasets; in green, STs found in the QUERY dataset only; in blue, the primary ST founder of the clonal complexes; in yellow, the subgroup founder of the clonal complexes; and in black, all other STs.

From the comparison with current human* E. coli* STs in China (human ST source: [http://enterobase.warwick.ac.uk/species/ecoli/search_strains?query=st_search]), all the isolates except for ST3705, ST716, ST3014, ST7588, ST398, ST1434, ST181, ST5143, ST3782, and ST3849 and human source* E. coli* presented STs with very close clonal relationships, such as SLVs, DLVs, or TLVs. The STs were distributed in nine clonal complexes: CC10, CC88, CC46, CC542, CC4995, CC206, CC224, CC58, and CC710. Human source STs, such as ST10, ST46, ST361, and ST2179, were also found in mink* E. coli* isolates (Figure 3).

As shown in the heatmap of the phylogenetic tree, most of the same ST strains were concentrated in the phylogenetic tree. The strains of the CC46 and CC176 clonal complexes were centrally distributed in the phylogenetic tree. The strains of the CC10 clonal complex were distributed in two regions in the phylogenetic tree.

There were significant differences between the drug resistance spectrum composition and drug resistance gene carriage status among most of the same ST strains or strains of the same clonal complex in the phylogenetic tree. There were no significant differences, or the differences were comparatively close between the drug resistance spectrum composition and drug resistance gene carriage status among the strains with close genetic relationships, such as 14-ST540 and 17-ST206.

**Figure 4:**
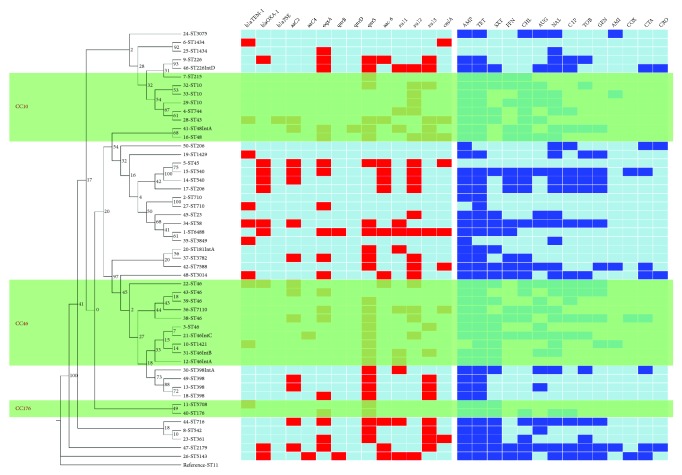
Diversity profiles of the phylogenetic tree, drug resistance genes, antimicrobial resistance, and Class 1 integron structure.* Note*. Dendrogram of seven allele sequence types from 50* E. coli* isolates from minks. ST11 is included as the reference. The three shaded parts in the figure represent three clonal complexes, CC10, CC46, and CC176 from top to bottom. The unit information is the number of isolated strains followed by the ST type and Class 1 integron structure IntA, dfrA27+aadA2+qnrA; IntB, dfrA17+aadA5; IntC, dfrA12+aadA2; or IntD, dfrA1+aadA1. (1) The 14 detected resistance genes were shown in the left matrix: a red square indicates that the strain carries the resistance gene, and a light blue square indicates that the resistance gene is not detected. (2) Antibiotics used in this experiment were shown in the right matrix: a blue square indicates that the strain is resistant to the antibiotic, and a light blue square indicates that the strain is not resistant.

There was no correlation between strains carrying different integrons or serotypes and their sequence type (Figure 4).

## 4. Discussion

In this study, 50* E. coli* strains were recovered from minks in the Zhucheng region. The serotyping results showed that the most prevalent serovars were EHEC and ETEC, while there was no EPEC found in our study. It was worth mentioning that the O104:H4 EHEC strain was detected in our study. Some serotypes of EHEC carry Shiga toxins, especially* E. coli* O157, which commonly carries Shiga toxins (stx1 and stx2). Additionally, what makes this pathogen more important is that it is even capable of expressing stx1 and stx2 under the VBNC state according to the latest study [36]. In view of the relatively poor sanitation conditions in livestock breeding environments, the EHEC O104:H4 strain may be a potential pathogenic factor in mink farms. We should pay more attention to EHEC O104:H4 in the mink breeding industry and control measures should also be implemented.

In the antimicrobial susceptibility test, most strains were found to be susceptible to cefoxitin and amikacin, which was consistent with the results of a previous report focused on red foxes in Portugal [37] but was not consistent with a report on minks in Denmark, which may be due to differences in geography and rearing conditions [38]. Most isolates were resistant to ampicillin (46/50, 92.0 %) and tetracycline (45/50, 90.0 %), which was similar to a report on minks in Denmark [38, 39], but the percentage of resistance to ampicillin and tetracycline was much higher than that of Pedersen's study (59.1 % and 75.5 %) [38]. Our study showed that 43 isolates (43/50, 86.0 %) were MDR, which was much higher than the MDR rate in minks in Denmark (60 %) [38].

Integrons are natural, highly efficient recombination and expression systems that can capture genes as part of genetic elements known as gene cassettes [40]. In recent studies, Class 1 integrons were investigated in animal, water, and human stool samples [41–44]; however, there have been few reports focusing on the prevalence of Class 1 integrons in strains of* E. coli* from minks. In this study, we investigated the occurrence and cassette region composition of Class 1 integrons among 50* E. coli *isolates from minks. Among the 50 isolates recovered from minks, 7 isolates (7/50, 14.0 %) carried Class 1 integrons, which was similar to that (16.0 %) of a previous study in sheep [6] but was lower than that of rabbits and cattle [45, 46]. These different results may be due to differences in species, environments and regions. Four transferable DHFR genes were detected in 50 isolates,* dfrA1*,* dfrA12*,* dfrA17*, and* dfrA27*; these gene cassettes were encoded by the trimethoprim resistance gene. Three transferable AAD genes were detected in 50 isolates,* aadA1*,* aadA2*, and* aadA5*; these gene cassettes were encoded by the aminoglycoside resistance gene. In addition,* qnrA* was encoded by the quinolone resistance gene. Four kinds of gene cassette arrays,* dfrA27* +* aadA2* +* qnrA*,* dfrA17* +* aadA5*,* dfrA12* +* aadA2,* and* dfrA1* +* aadA1*, were identified in this study. These integrons were found not only in* Salmonella* [47, 48] and* E. coli* [49, 50] from chickens, ducks, and pigs, but also in* Klebsiella pneumoniae* [51] and* Staphylococcus aureus* [52] isolated from China, suggesting that gene cassette arrays can be transferred to different species of bacteria with the horizontal movement of integrons and then transmitted to different species. The horizontal transfer of integrons may have an important effect on the clinical use of antibiotics. Of note, Class 1 integrons are usually associated with MDR* E. coli *isolates, which is consistent with the results of this study.

A wide variety of resistance genes were found in nonpathogenic* E. coli *strains from minks. The inclusion of some resistance genes inside integrons may explain the spread of antibiotic resistance among minks in the Zhucheng area. Among the three types of *β*-lactamase resistance genes detected in our study, 20.0 % of the* E. coli* isolates carried *bla*_TEM_ and *bla*_OXA_, whereas 92.0 % of the isolates were resistant to ampicillin; this may be related to the expression level of *bla*_TEM_ and *bla*_OXA_ genes and requires further study. Our data showed that 40.0 % of the* E. coli* isolates carried* sul2,* 32.0 % of the* E. coli* isolates carried* sul3*, 24.0 % of the* E. coli* isolates carried* sul1, *and* 58.*0 % of the isolates were resistant to sulfamethoxazole. The detection rates of *β*-lactamase and sulfamethoxazole resistance genes in our study are similar to those in red foxes in northern Portugal [37]; however, there were some slight differences; for instance, both *bla*_TEM_ and *bla*_OXA_ were detected in our study, but only *bla*_TEM_ was detected in the previous study [37]. For the sulfamethoxazole resistance genes,* sul1, sul2, *and* sul3,* were all detected in our study, but only* sul1* was detected in the red fox study [37]. These differences may be related to the differences in regions and species.

In our study, the quinolone genes (*qnrS*,* qnrB*,* qnrD,* and* oqxA*) were detected, alone or in combination in quinolone-resistant isolates, revealing that the frequent occurrence of quinolone genes may be associated with high antibiotic resistance rates to quinolone. Nevertheless, our results showed that there was no tetracycline resistance gene among mink* E. coli* isolates, which was not consistent with the resistance rate of tetracycline (90.0 %).

The MLST method provides a scalable typing system that reflects the population and evolutionary biology of bacteria and makes valid comparisons between results from different laboratories possible. MLST applies neutral or slowly accumulating genetic variations in housekeeping genes, which are not affected by the rapid evolution detected within genes encoding proteins that influence survival in a particular niche [53–55]. MLST analyses rely on the sequencing of seven housekeeping genes for each* E. coli* isolate. The sequences are compared using an online database (http://www.mlst.net) that attributes the allelic profile and performs the concatenation leading to the sequence type. MLST results are accurate and reproducible between laboratories and, over time, provide detailed information on the overall epidemiology of the organism [56, 57]. STs are grouped in clonal complexes (CCs) of isolates that share five to seven alleles with another sequence type in the group [58]. Therefore, the clonal relationship of* E. coli* from minks was studied by MLST, and a phylogenetic tree was constructed based on the strain MLST data to understand the apparent partial characteristics of strains with different genetic relationships and the differences in drug resistance gene carriage.

In our study, 31 previously known STs were found, of which 18 STs (10, 23, 43, 46, 48, 58, 181, 206, 215, 226, 398, 716, 744, 1421, 1434, 3075, 5708, and 7588) were clustered in 10 CCs (10, 23, 46, 155, 168, 206, 226, 398, 467, and 522) (Figure 2), and the remaining 13 STs did not have CCs in the BURST algorithm. Ten previously known STs (10, 43, 48, 215, 744, 1421, 46, 7110, 176, and 5708) were found in 31 STs of 50* E. coli* strains, which were clustered in three CCs (10, 46, and 176) (Figure 2). This finding differs from a previous study focused on* E. coli* of rabbits performed in Shandong Province [45]; these differences may indicate that, compared with other animals such as rabbits,* E. coli* strains from minks in Shandong Province have greater ST diversity and more CCs. The most frequently detected ST was ST46 (8/50, 16%) in the study. Compared with other reports, there were significant differences in STs and dominant STs detected from different regions and species. For example, ST302 was the dominant ST in rabbits in Shandong Province [45], ST710 was the dominant ST in non-O157 Shigatoxin-producing* Escherichia coli *(STEC) strains isolated from different resources in China [59], and ST101 was the dominant ST in edible animals in China [60]. This indicates that the genotypes of* E. coli* are widely distributed, and the dominant ST is obvious different among different regions. The reasons for this phenomenon may be related to the cloning and transmission of* E. coli* strains. Compared with 340 STs of human* E. coli* from China, we found that 17 STs of mink origin differed from the human STs by one allele and formed 9 clones (Figure 3). Four STs (10, 46, 361, and 2179) were the same as human STs (Figure 3). CC10 showed an interesting and unexpected result because the cluster contained 48 STs (8 from animals and 41 from humans). ST10 is a strong possible common ancestor candidate for CC10. ST48 from mink is a CC10 subgroup founder connecting five different STs (3127, 3932, 2739, 2434, and 4082) from human sources. These results show that some mink-derived* E. coli* are closely related to human* E. coli *(in the Chinese population), and these STs might be related to some diseases (based on http://enterobase.warwick.ac.uk/species/ecoli/search_strains?Query=st_search). To prevent threats to public health, therefore, continuous monitoring and detection of* E. coli* in minks are necessary.

By studying the results of the phylogenetic tree, we found that the strains of the same ST, such as ST10, ST46, and ST1434, were distributed in the same region on the phylogenetic tree and had the closest relationship with each other (Figure 4). Most strains of the same CC were concentrated in phylogenetic trees, but ST48 in CC10 was far from other strains in CC10. We speculated that the difference in the adK gene between ST48 and ST10 may be the cause of this distribution. Antibiotic resistance profiles and resistance gene profiles of ST genotypes with different genetic relationships were observed. We can conclude that the distribution of different serotypes and integron-carrying strains in phylogenetic trees has no obvious regularity. From the phylogenetic tree as a whole, there were clear differences in the composition of the drug resistance spectrum and the types of drug resistance genes carried by the closely related strains, but there were also some closely related strains with drug resistance spectrum composition and types of drug resistance gene carried that were very similar (17-ST206 and 14-ST540) (Figure 4). These strains have a high level of genetic similarity.

## 5. Conclusions

In summary, we conducted an epidemiological survey of* E. coli* on mink farms in Zhucheng. Our results showed that* E. coli* was highly prevalent in minks. The reported pathogenic serotypes and MDR* E. coli* strains contained abundant types of drug resistance genes and a certain number of gene cassettes.* E. coli* isolated from mink origin had similar genes, and some strains had high genetic similarity with* E. coli* strains isolated from humans in China. Therefore, the relevant breeding enterprises should manage and prevent these pathogenic bacteria to avoid harm to public health.

## Figures and Tables

**Table 1:** Primers used in the PCRs carried out in this study.

Primer	Sequence (5′-3′)	Target	PCR product size (bp)
*Class 1 Integron*			
*Hep58*	*TCA TGG CTT GTT ATG ACT GT*	*Class 1 integron variable region*	Variable
*Hep59*	GTA GGG CTT ATT ATG CAC GC		
*β-Lactamases*			
*TEM-F*	ATAAAATTCTTGAAGACGAAA	*bla*_*TEM*_	643
*TEM-R*	GACAGTTACCAATGCTTAATC		
*SHV-F*	TTATCTCCCTGTTAGCCACC	*bla*_*SHV*_	860
*SHV-R*	GATTTGCTGATTTCGCTCGG		
*PSE-F*	TAGGTGTTTCCGTTCTTG	*bla*_*PSE*_	150
*PSE-R*	TCATTTCGCTCTTCCATT		
*OXA-F*	TCAACTTTCAAGATCGCA	*bla*_*OXA*_	591
*OXA-R*	GTGTGTTTAGAATGGTGA		
*CMY-2-F*	ACGGAACTGATTTCATGATG	*bla*_*CMY-2*_	714
*CMY-2-R*	GAAAGGAGGCCCAATATCCT		
*Tetracyclines*			
*tetA-F*	GCTACATCCTGCTTGCCTTC	*tetA*	211
*tetA-R*	CATAGATCGCCGTGAAGAGG		
*tetB-F*	TTGGTTAGGGGCAAGTTTTG	*tetB*	391
*tetB-R*	GTAATGGGCCAATAACACCG		
*Plasmid-mediated quinolones*			
*qnrA-F*	ATTTCTCACGCCAGGATTTG	*qnrA*	519
*qnrA-R*	GATCGGCAAAGGTTAGGTCA		
*qnrB-F*	GATCGTGAAAGCCAGAAAGG	*qnrB*	513
*qnrB-R*	ACGATGCCTGGTAGTTGTCC		
*qnrC-F*	GGTTGTACATTTATTGAATC	*qnrC*	666
*qnrC-R*	TCCACTTTACGAGGTTCT		
*qnrD-F*	AGATCAATTTACGGGGAATA	*qnrD*	984
*qnrD-R*	AACAAGCTGAAGCGCCTG		
*qnrS-F*	ACGACATTCGTCAACTGCAA	*qnrS*	417
*qnrS-R*	TAAATTGGCACCCTGTAGGC		
*aac(6*′*)-Ib-F*	TTGCGATGCTCTATGAGTGGCTA	*aac(6*′*)-Ib-cr*	482
*aac(6*′*)-Ib-R*	CTCGAATGCCTGGCGTGTTT		
*oqxA-F*	GATCAGTCAGTGGGATAGTTT	*oqxA*	670
*oqxA-R*	TACTCGGCGTTAACTGATTA		
*Chloramphenicols*			
*cmlA-F*	TGCCAGCAGTGCCGTTTAT	*cmlA*	900
*cmlA-R*	CACCGCCCAAGCAGAAGTA		
*stcM-L*	CACGTTGAGCCTCTATATGG	*floR*	890
*stcM-R*	ATGCAGAAGTAGAACGCGAC		
*Sulphonamides*			
*sul1-F*	CTTCGATGAGAGCCGGCGGC	*sul1*	238
*sul1-F*	GCAAGGCGGAAACCCGCGCC		
*sul2-F*	GCGCTCAAGGCAGATGGCATT	*sul2*	793
*sul2-F*	GCGTTTGATACCGGCACCCGT		
*sul3-R*	AGATGTGATTGATTTGGGAGC	*sul3*	443
*sul3-R*	TAGTTGTTTCTGGATTAGAGCCT		
*Aminoglycosides*			
*aac(3)-I-F*	ACCTACTCCCAACATCAGCC	*aac(3)-I*	528
*aac(3)-I-R*	ATATAGATCTCACTACGCGC		
*aac(3)-II-F*	ACTGTGATGGGATACGCGTC	*aac(3)-II*	482
*aac(3)-II-R*	CTCCGTCAGCGTTTCAGCTA		
*aac(3)-III-F*	CACAAGAACGTGGTCCGCTA	*aac(3)-III*	185
*aac(3)-III-R*	AACAGGTAAGCATCCGCATC		
*aac(3)-IV-F*	CTTCAGGATGGCAAGTTGGT	*aac(3)-IV*	286
*aac(3)-IV-R*	TCATCTCGTTCTCCGCTCAT		
*Ant(2*′*)-F*	ATGTTACGCAGCAGGGCAGTCG	*Ant(2*′)	187
*Ant(2*′*)-R*	CGTCAGATCAATATCATCGTGC		

**Table 2:** Diversity profiles of *E. coli* isolates based on MLST, serovar, and Class 1 integron structure.

ST type	Allele Profile^a^	ST Complex^b^	No. (n=50)^c^	Serovar^d^	Class 1 Integron Structure
ST10	10,11,4,8,8,8,2	ST10 Cplx	29		-
ST10	10,11,4,8,8,8,2	ST10 Cplx	32		-
ST10	10,11,4,8,8,8,2	ST10 Cplx	33		-
ST23	6,4,12,1,20,13,7	ST23 Cplx	45		-
ST43	24,11,4,8,8,8,2	ST10 Cplx	28		-
ST46	8,7,1,8,8,8,6	ST46 Cplx	3		-
ST46	8,7,1,8,8,8,6	ST46 Cplx	12	O20:K71	-
ST46	8,7,1,8,8,8,6	ST46 Cplx	21		*dfrA27+aadA2+qnrA*
ST46	8,7,1,8,8,8,6	ST46 Cplx	22		*dfrA12+aadA2*
ST46	8,7,1,8,8,8,6	ST46 Cplx	31		*-*
ST46	8,7,1,8,8,8,6	ST46 Cplx	38		*dfrA17+aadA5*
ST46	8,7,1,8,8,8,6	ST46 Cplx	39		*-*
ST46	8,7,1,8,8,8,6	ST46 Cplx	43		*-*
ST48	6,11,4,8,8,8,2	ST10 Cplx	16	O20:K71	*-*
ST48	6,11,4,8,8,8,2	ST10 Cplx	41		*-*
ST58	6,4,4,16,24,8,14	ST155 Cplx	34		*dfrA27+aadA2+qnrA*
ST176	10,4,5,1,8,8,2	-	40		*-*
ST181	8,11,4,8,7,8,6	ST168 Cplx	20		*-*
ST206	6,7,5,1,8,18,2	ST206 Cplx	17		*dfrA27+aadA2+qnrA*
ST206	6,7,5,1,8,18,2	ST206 Cplx	50	O104:H4	*-*
ST215	10,11,4,8,8,18,2	ST10 Cplx	7		*-*
ST226	10,27,5,8,8,7,2	ST226 Cplx	9	O152:K?	*-*
ST226	10,27,5,8,8,7,2	ST226 Cplx	46		*-*
ST361	10,99,5,91,8,7,2	-	23		*dfrA1+aadA1*
ST398	64,7,1,1,8,8,6	ST398 Cplx	13	O104:H4	*-*
ST398	64,7,1,1,8,8,6	ST398 Cplx	18		*-*
ST398	64,7,1,1,8,8,6	ST398 Cplx	30		*-*
ST398	64,7,1,1,8,8,6	ST398 Cplx	49		*dfrA27+aadA2+qnrA*
ST540	6,7,57,1,8,8,2	-	5		*-*
ST540	6,7,57,1,8,8,2	-	14		*-*
ST540	6,7,57,1,8,8,2	-	15		*-*
ST542	112,11,5,12,8,8,86	-	8	O104:H4	*-*
ST710	6,153,4,91,7,8,6	-	2		*-*
ST710	6,153,4,91,7,8,6	-	27		*-*
ST716	10,7,4,140,8,8,2	ST10 Cplx	44		*-*
ST744	10,11,135,8,8,8,2	ST10 Cplx	4		*-*
ST1421	8,7,1,8,8,8,2	ST46 Cplx	10		*-*
ST1429	6,4,109,1,8,8,6	-	19		-
ST1434	10,11,5,8,7,8,6	ST10 Cplx	6		-
ST1434	10,11,5,8,7,8,6	ST10 Cplx	25		-
ST2179	9,65,5,18,11,8,6	-	47		-
ST3014	303,41,1,8,8,8,6	-	48		-
ST3075	10,23,109,8,270,8,2	ST522 Cplx	24		-
ST3782	64,196,188,83,24,8,6	-	37		-
ST3849	1,4,44,9,11,2,7	-	35		-
ST5143	332,40,354,13,36,28,29	-	26	O9:K9	-
ST5708	10,4,5,8,8,8,2	ST10 Cplx	11		-
ST6488	6,4,33,16,11,8,7	-	1		-
ST7110	8,7,1,8,8,8,508	-	36		-
ST7588	8,7,4,8,8,512,6	ST467 Cplx	42		-

*Note.*  ^a^Allele number for adK, fumC, gyrB, icD, mdH, purA, and recA, respectively (one for each ST). ^b^ST complex of ST type. ^c^The number corresponds to the positive samples among the total of 50. ^d^O152:K?, Enteroinvasive *E. coli* (EIEC); O9:K9 and O20:K17, Enterotoxigenic *E. coli* (ETEC); O104:H4, Enterohemorrhagic *E. coli* (EHEC).

## Data Availability

The data used to support the findings of this study are available from the corresponding author upon request.
